# Mind the brain gap: The worldwide distribution of neuroimaging research on adolescent depression

**DOI:** 10.1016/j.neuroimage.2021.117865

**Published:** 2021-02-14

**Authors:** Lucas Battel, Fernanda Cunegatto, Anna Viduani, Helen L. Fisher, Brandon A. Kohrt, Valeria Mondelli, Johnna R. Swartz, Christian Kieling

**Affiliations:** a Department of Psychiatry, Universidade Federal do Rio Grande do Sul (UFRGS), Child & Adolescent Psychiatry Division, Hospital de Clínicas de Porto Alegre (HCPA), Rua Ramiro Barcelos, 2350 – 400N, Porto Alegre, RS 90035003, Brazil; b King’s College London, Social, Genetic & Developmental Psychiatry Centre, Institute of Psychiatry, Psychology & Neuroscience, 16 De Crespigny Park, London SE5 8AF, United Kingdom; c ESRC Centre for Society and Mental Health, Virginia Woolf Building, 22 Kingsway, London WC2B 6NR, United Kingdom; d Division of Global Mental Health, Department of Psychiatry, School of Medicine and Health Sciences, The George Washington University, 2120L St NW, Ste 600, Washington DC 20037, United States; e King’s College London, Department of Psychological Medicine, Institute of Psychiatry, Psychology & Neuroscience, Cutcombe Road, London SE5 9RT, United Kingdom; f National Institute for Health Research Mental Health Biomedical Research Centre, South London and Maudsley NHS Foundation Trust and King’s College London, London, United Kingdom; g Department of Human Ecology, University of California, Davis, One Shields Ave, Davis, CA 95616, United States; h Department of Psychiatry, UFRGS, Child & Adolescent Psychiatry Division, HCPA, Rua Ramiro Barcelos, 2350 – 400N, Porto Alegre, RS 90035003, Brazil

**Keywords:** Adolescence, Depression, Neuroimaging, Income, Inequality, Developing countries

## Abstract

Adolescents comprise one fourth of the world’s population, with about 90% of them living in low- and middle-income countries (LMICs). The incidence of depression markedly increases during adolescence, making the disorder a leading cause of disease-related disability in this age group. However, most research on adolescent depression has been performed in high-income countries (HICs). To ascertain the extent to which this disparity operates in neuroimaging research, a systematic review of the literature was performed. A total of 148 studies were identified, with neuroimaging data available for 4,729 adolescents with depression. When stratified by income group, 122 (82%) studies originated from HICs, while 26 (18%) were conducted in LMICs, for a total of 3,705 and 1,024 adolescents with depression respectively. A positive Spearman rank correlation was observed between country *per capita* income and sample size (r_s_ =0.673, *p* = 0.023). Our results support the previous reports showing a large disparity between the number of studies and the adolescent population per world region. Future research comparing neuroimaging findings across populations from HICs and LMICs may provide unique insights to enhance our understanding of the neurobiological processes underlying the development of depression.

## 1. Introduction

Children and adolescents account for one fourth of the world’s population, and most individuals in this age group – nine out of 10 youth under the age of 18 years – live in low- and middle-income countries (LMIC) (Kieling et al., 2011) as defined by the World Bank based on gross national income (GNI) *per capita* (World Bank, 2020). However, a striking disparity has been noted between the world distribution of the youth population and the scientific output in the overall field of child and adolescent mental health, with 90% of the publications on the topic originating from high-income countries (HIC), where only 10% of children and adolescents actually live (Kieling and Rohde, 2012).

The same disparity seems to operate in genomic and neuroimaging research, both of which have provided important contributions to the understanding of mental health disorders and specifically major depressive disorder (MDD), a leading cause of disease-related disability in adolescence (Thapar et al., 2012). In genomic research, analyses of published data reveal a large sampling bias in previous decades, with 96% of genomic studies performed with people of European ancestry (Bustamante et al., 2011). Despite the ongoing efforts to diversify research populations (Hindorff et al., 2018), recent data still suggest that non-Europeans make up less than 20% of genome-wide association studies (Popejoy and Fullerton, 2016). In MDD, the role of sociocultural factors in the etiology and clinical expression of the disease makes it compelling to aim for a balanced representation of people from diverse global settings. Further, there is growing evidence for cultural differences in social cognitive processes, which can be observed through neuroimaging (Han and Ma, 2014).

The field of neuroimaging research is of growing importance for the understanding of neural mechanisms associated with the onset of depression in adolescence (Kerestes et al., 2014). A recent bibliographic review has found that among the 100 most cited neuroimaging studies focusing on psychiatric disorders, only six were conducted by researchers in LMICs – China in all cases (Gong et al., 2019). Even considering the limited affordability of neuroimaging techniques in low resource settings (McLane et al., 2015), such imbalance brings into question the global representativeness of the available findings. Variation in socioeconomic context, culture, and peer environment are all associated with differences in adolescent neurocognitive processing and brain development (Foulkes and Blakemore, 2018). Moreover, there is wide variation in age menarche and adrenarche across populations globally, with age of onset later in many low- and middle-income countries (Worthman and Trang, 2018). Given the effects of puberty on brain development (Goddings et al., 2014), it would be important to understand how global variation in this timing affects population differences in adolescent mental health.

Although some research initiatives (e.g., ABCD) have focused more on cultural and environmental differences on adolescent brain development within a HIC (Zucker et al., 2018), our scientific understanding would be much improved by capturing the global diversity in culture and environments. Additionally, possible commonalities and differences in functional neuroimaging patterns in patients with MDD in HICs vs. LMICs will require considerable efforts to leverage discovery science in the latter, given that the available knowledge cannot be readily implemented in these understudied settings (Freeman et al., 2016; Stein and Wegener, 2017). Considering this scenario, we conducted a systematic review of the literature to compare the scientific output in neuroimaging research and the size of the adolescent population in countries classified into different income categories. We hope to make any existing disparities more evident, helping to shift future research resources to lower income areas and reduce potential limitations of the current literature in the field.

## 2. Methods

### 2.1. Search strategy

We performed a systematic search of MEDLINE and Web of Science databases to retrieve studies published in any language using specific terms for adolescence, depression, and neuroimaging (full search syntax can be found in the Supplementary Material 1). No specific timeframe was set. The search was conducted on January 1, 2020. The initial results were screened by two independent authors (AV and FC), who also independently reviewed the full text of selected manuscripts and extracted the data of interest. This process was overseen by a third author (LB). Unresolved discrepancies were discussed with a senior researcher (CK) to reach a final consensus. The systematic review was performed and reported according to the Preferred Reporting Items for Systematic Reviews and Meta-Analyses (PRISMA) guidelines (flow diagram in the Supplementary Material 2).

### 2.2. Inclusion and exclusion criteria

Original empirical studies published in peer-reviewed journals were included if they had an adolescent sample, based on a recent broad definition of this age period (i.e., age ranging from 10 to 24 years) (Sawyer et al., 2018), with clinical depression (defined by study author), provided a categorical or dimensional measure of MDD, and reported any form of magnetic resonance imaging (MRI). Studies evaluating bipolar depression and those without a group of adolescent patients with unipolar depression at the time of MRI scanning were excluded from the analyses. This also applied to longitudinal studies, which were excluded if no scans of adolescents with a depressive episode were reported.

### 2.3. Variables of interest and data analysis

The following variables were recorded for each study: first and corresponding authors, year of publication, imaging technique – structural MRI (sMRI), resting-state functional MRI (rs-fMRI), task-based functional MRI (tb-fMRI), magnetic resonance spectroscopy (MRS), or diffusion tensor imaging (DTI), – country where the sample was enrolled, sample size, age, and sex. All countries were classified as HIC or LMIC, as defined by the World Bank based on the most recent data (World Bank, 2020). Following the same classification, LMIC was further subdivided into upper-middle-, lower-middle-, and low-income (World Bank, 2020). Up-to-date information on total adolescent population (World Bank, 2018b) and GNI (World Bank, 2018a) were also extracted from World Bank data at the time of analysis. For statistical analyses, only participants with depression (as defined by authors) in each study were considered, to avoid confounders in samples with other major psychopathology categories.

Data analyses included a description of the overall sample characteristics for each country income group with comparison of age and sex, using *t*-test for quantitative variables and chi-square tests for categorical variables. Spearman rank correlations were calculated for pairwise comparisons of continuous variables to account for non-normal distributions. For a better assessment of the individuals included in each study relative to the population of adolescents in each country, an adjusted index of representativeness was generated. This index, obtained by dividing the number of study participants by the total adolescent population of each country, provided an objective comparison among the countries, with correction of possible outliers in countries with disproportionately small or large populations.

## 3. Results

**Fig. 1.**
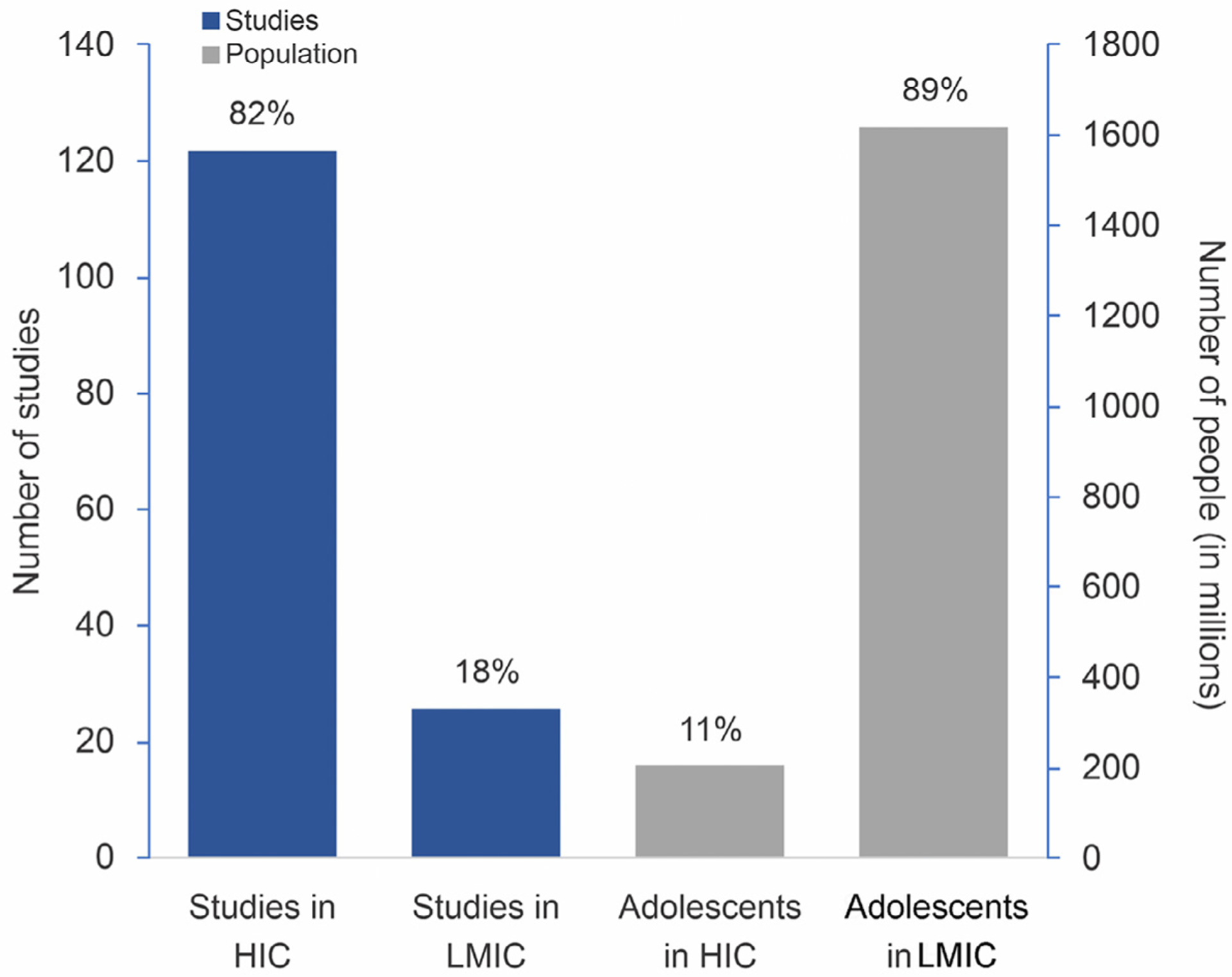
Proportion of neuroimaging studies performed in high-income vs. low-income countries and proportion of adolescents in the population of each country income group. Population data from the most current World Bank database (World Bank, 2018b). HICs, High income countries; LMICs, low- and middle-income countries.

Characteristics of the 148 studies meeting the inclusion criteria for analysis are presented in Table 1. Neuroimaging data were available on 4729 individuals with depression. Considering country income, 122 (82%) studies including 3705 adolescents (mean 30; min 5, max 123) originated from HICs, while 26 (18%) studies including 1024 adolescents (mean 39; min 14, max 108) were performed in LMICs. The mean number of study participants was statistically higher in LMICs (*p*<0.001). Table 2 contains descriptive statistics of the studies according to country. A full list of included studies is available in Supplementary Material 3. Fig. 1 shows the proportion of studies performed in HICs vs. LMICs and the distribution of the adolescent population according to country income group.

**Fig. 2.**
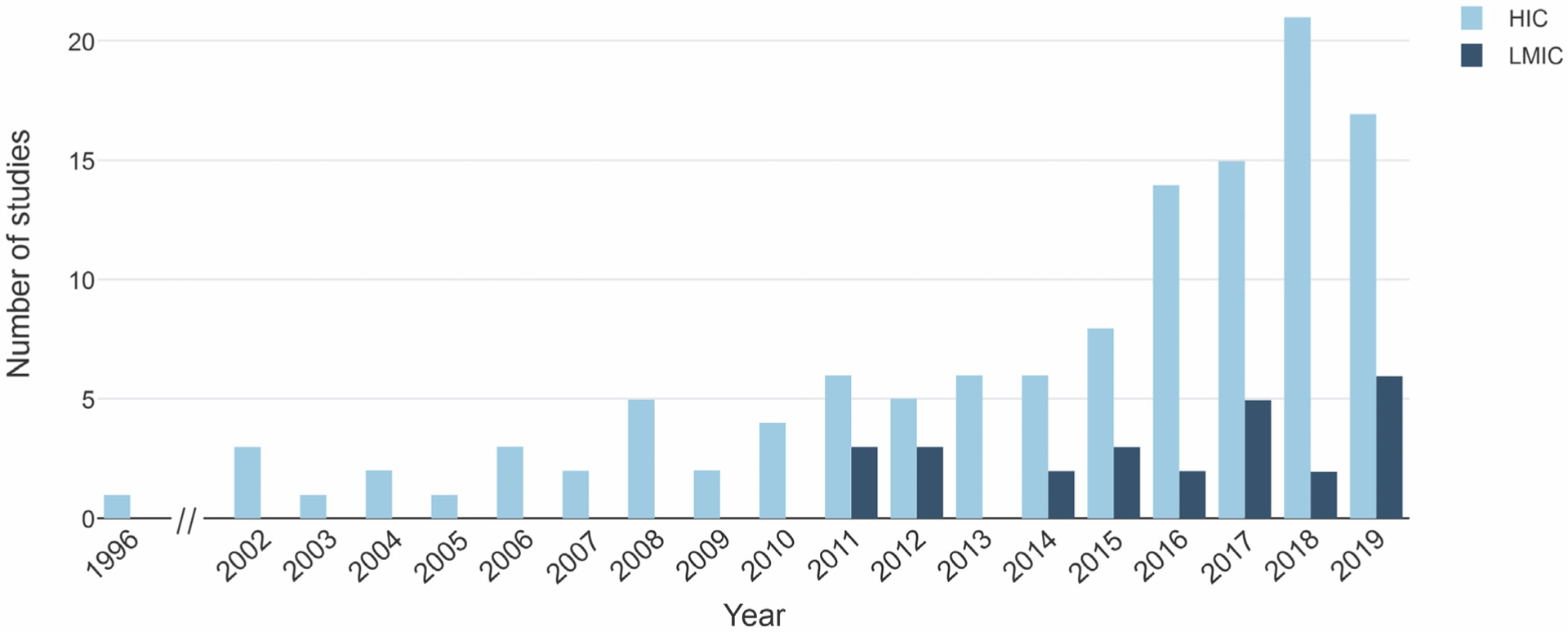
Number of neuroimaging publications on adolescent depression per year according to country income group. HICs, high-income countries; LMICs, low- and middle-income countries.

Studies from 8 HICs were included. The largest number of publications (81, 55% of all studies) came from the United States. Australia and Canada came in second and third, with 12 (8%) and 9 (6%) studies respectively. In terms of sample size, the United States was also first, with 2430 youth with depression, accounting for just over half the adolescents studied worldwide. Only three LMICs had studies included in the present analyses: China (23 studies, nMDD=859), Brazil (2 studies, nMDD=85), and Romania (1 study, nMDD=80). All these LMICs can be further classified as upper-middle-income based on the World Bank definition (World Bank, 2020). There were no studies focusing on adolescents with depression from lower-middle- or low-income countries. The number of studies published per year according to country income group is shown in Fig. 2. While the first neuroimaging study on adolescent depression came from a HIC in 1996, the first LMIC publication on this topic was not until 2011. There was a positive correlation between sample size and year of publication (r_s_ =0.326, *p<*0.001).

Regarding neuroimaging modality (Table 1), the HIC group had a higher proportion of studies assessing sMRI (57%; *χ*^2^ = 8.0, *p* = 0.005) and tb-fMRI (43%; *χ*^2^ = 6.8, *p* = 0.009). In contrast, proportionally more studies from LMICs used rs-fMRI (58%; *χ*^2^ = 16.0, *p<*0.001). Finally, MRS and DTI were the least studied techniques overall, with 14 and 11 studies, respectively.

**Fig. 3.**
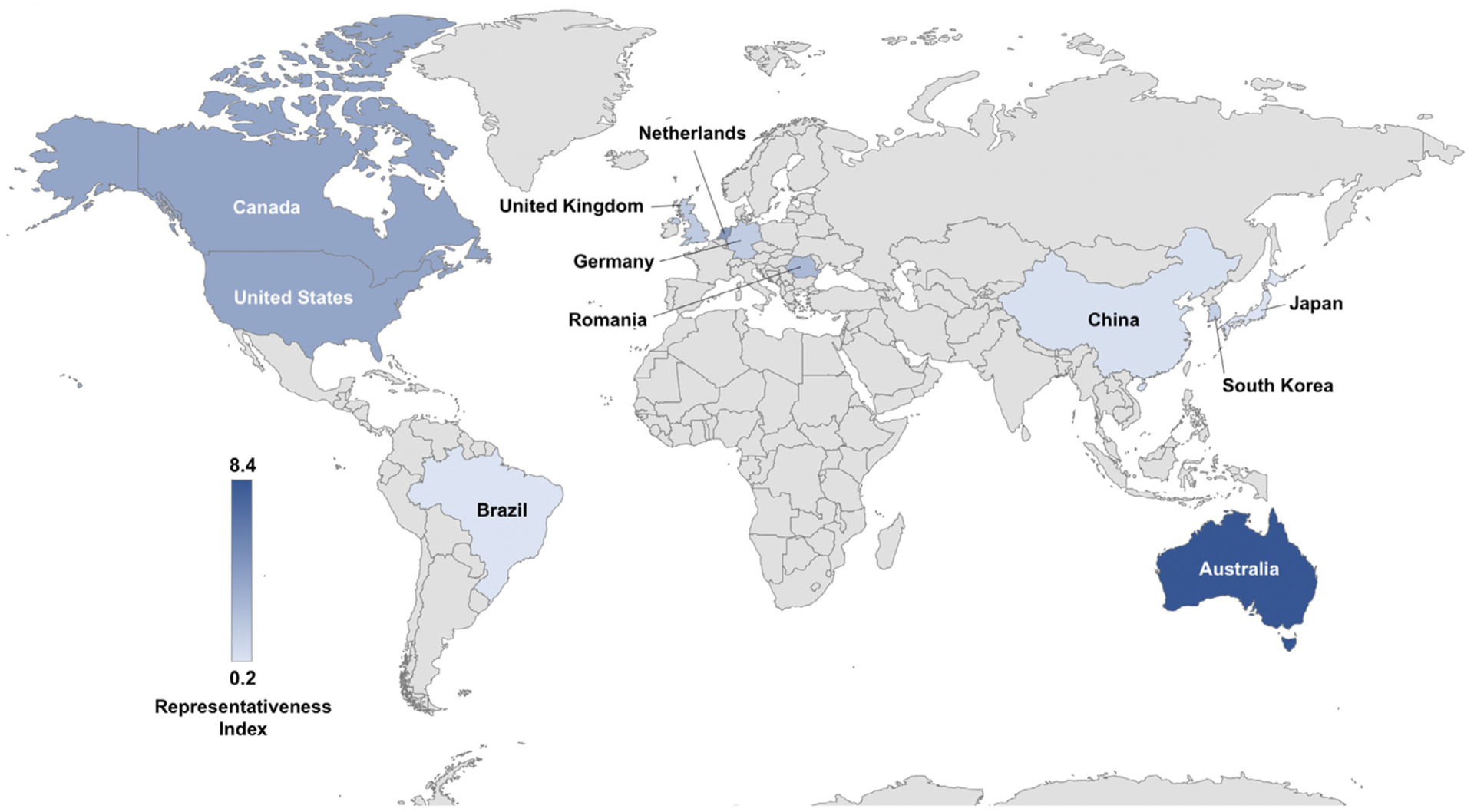
Representativeness of MRI study samples in youth depression worldwide – the index represents the number of participants in MRI studies per 100,000 adolescents in each country, showing that most adolescents included were from high-income countries.

Fig. 3 shows a representativeness index (number of MDD subjects scanned/100,000 adolescents in the population) for each country on a world map. A higher index means a higher representation of the adolescent population from that particular country, i.e., a higher proportion of adolescents from that country were studied. We found a direct correlation between the GNI *per capita* and the adjusted sample size of the included studies (r_s_ =0.673, *p* = 0.023). Moreover, this correlation remained significant while controlling for year of publication of each study (r_s_ =0.636, *p<*0.05). Further, using the World Bank data, we estimated that 1.4 billion adolescents worldwide (78% out of a total of 1.8 billion) lived in countries without any neuroimaging studies on youth depression.

## 4. Discussion

Much has been learnt about the neurobiology of depression in adolescence over the past two decades. A large number of studies have been published on this matter, encompassing diverse brain imaging techniques and sample characteristics. However, as shown in the present study, there is a vast gap between countries regarding the distribution of the adolescent population *vis-à-vis* the performance of neuroimaging research, with more research being performed in countries with smaller adolescent populations. A decade ago, this phenomenon was described as the 10–90% divide in the context of research into treatment of adolescent mental health disorders (Kieling et al., 2011). At that time, it became evident that 90% of the studied individuals only accounted for 10% of the worldwide population in terms of income and socioeconomic status. That prompted a call for action to increase research investment in LMICs, where the majority of adolescents actually live (Kieling et al., 2011).

In the present study, only a slight improvement was observed regarding the number of studies and the sample sizes when comparing HICs vs. LMICs in terms of neuroimaging research. Of 148 neuroimaging studies in adolescent samples with depression, only 26 (18%) were conducted in LMICs. Further, only three countries figured in this group: Brazil, China, and Romania, all subcategorized as upper-middle-income. No studies from lower-middle or low-income nations were found. While the mean number of adolescents with depression per study was higher in the LMIC group, this is likely related to the HIC group having earlier, smaller studies, as year of publication was also correlated to number of adolescents with MDD.

One likely reason for the low representativeness of LMICs in neuroimaging research on adolescent depression is the high cost of neuroimaging technology, which limits access to such investigational modalities. This is supported by the positive correlation between the overall number of adolescents studied per country and the gross national income *per capita*. Further, the small number of research groups working with neuroimaging methods in lower income countries hinders the diffusion of knowledge, especially involving complex and costly research activities. On the bright side, three studies from LMICs, namely China (nMDD=108) (Chang et al., 2018), Romania (nMDD=80) (Hogea et al., 2017), and Brazil (nMDD= 56) (Pan et al., 2017), attest to the feasibility of conducting larger neuroimaging studies in countries with more limited resources. Further, a collaborative research initiative is ongoing in Brazil, with preliminary neuroimaging data published for 29 adolescents with depression (Battel et al., 2020) and plans for further publications now that a total sample of 50 adolescents with depression has been collected.

Finally, our review is not without limitations. First, only the MEDLINE and Web of Science databases were searched in our systematic review. Even though these are the most widely used databases across the globe for indexing neuroimaging studies, some studies, especially small ones, may have been missed. Second, conference abstracts were not specifically searched to be included in our analyses. These aspects may have skewed the results towards the HIC group, since the publication step after data collection also imposes several additional difficulties for LMICs, including the cost of publication. Third, socioeconomic data from the included studies was largely unavailable in the publications and could not be analyzed. We assume that included samples from HICs have a higher mean income than samples from LMICs. However, a direct comparison with actual sample income assessed at the individual level would yield a more accurate representation of our findings. Fourth, our review only included MRI studies and did not include less expensive approaches such as EEG. An important question for future research is to determine whether other imaging approaches are more common in LMICs. Fifth, there is insufficient data in the current literature to allow for the generalization of our findings in terms of clinical implications. If differences exist between distinct populations, clinical studies will be necessary to assess the impact of such differences on prevention or treatment strategies.

## 5. Conclusions

As previously evidenced in the adolescent psychiatric literature, there is an important divide between where neuroimaging studies are conducted and where most of the affected population lives. The promise of cultural neuroscience will require that research be conducted in diverse settings (Choudhury, 2010). There is, therefore, a pressing need for larger research investments in LMICs. Finally, considering that depression has been inversely associated with socioeconomic status and educational levels in both high-income and low- and middle-income settings, differences in the neuroimaging patterns observed in patients with MDD in LMICs versus HICs are possible. Thus, the comparison of neuroimaging findings across populations from such nations may provide unique insights to enhance our understanding of the neurobiological processes involved in the development of depression in the first decades of life.

## Supplementary Material

Supplementary1

Supplementary2

Supplementary3

## Figures and Tables

**Table 1:** Study characteristics per country income group.

	HICs *n* = 122	LMICs *n* = 26
Mean age, years (SD)	17.0 (4.7)	18.9 (4.4)*
Female sex (%)	60.9	57.8
MRI modality, n (%)		
sMRI	70 (57)	7 (27)**
rs-fMRI	24 (20)	15 (58)*
tb-fMRI	52 (43)	4 (15)**
MRS	12 (10)	2 (8)
DTI	9 (7)	2 (8)

HICs, high-income countries; LMICs, low- and middle-income countries; MDD, major depressive disorder; MRI, magnetic resonance imaging; fMRI, functional MRI; sMRI, structural MRI; rs-fMRI, resting-state fMRI; tb-fMRI, task-based fMRI; MRS, magnetic resonance spectroscopy; DTI, diffusion tensor imaging. MRI modality% refers to each group; some studies had more than one modality.

* *p*<0.001.

** *p<*0.05.

**Table 2:** Descriptive characteristics of included participants by country.

Country	Studies (n)	MDD (n)	% female	Mean age, years (SD)	sMRI	rs-fMRI	tb-fMRI	MRS	DTI
Australia	12	394	56	18.8 (3.2)	6	4	5	1	0
Brazil	2	85	66	13.1 (2.1)	0	2	0	0	0
Canada	9	245	51	19.4 (1.3)	5	2	2	2	0
China	23	859	57	19.9 (4.0)	7	13	4	1	2
Germany	5	187	75	19.4 (3.5)	5	1	2	0	0
Japan	1	27	37	18.2 (0.4)	1	0	1	0	0
Netherlands	5	136	76	15.7 (0.2)	4	1	1	0	2
Romania	1	80	N/A	13.8 (4.0)	0	0	0	1	0
South Korea	4	104	64	20.6 (3.2)	2	2	0	0	0
UK	5	182	73	18.2 (3.4)	3	1	3	0	0
USA	81	2430	60	15.9 (5.1)	44	13	38	9	7
Total	148	4729	60	17.4 (4.7)	77	39	56	14	11

MDD (n), number of included depressed individuals; MRI, magnetic resonance imaging; fMRI, functional MRI; sMRI, structural MRI; rs-fMRI, resting-state fMRI; tb-fMRI, task-based fMRI; MRS, magnetic resonance spectroscopy; DTI, diffusion tensor imaging. N/A, not available in the original study.

## References

[R1] Battel L, Swartz J, Anes M, Manfro PH, Rohde LA, Viduani A, Mondelli V, Kieling C, 2020. Neuroimaging adolescents with depression in a middle-income country: feasibility of an fMRI protocol and preliminary results. Braz. J. Psychiatry 42, 6–13. 31389498 10.1590/1516-4446-2019-0508 PMC6986476

[R2] Bustamante CD, Burchard EG, De La Vega FM, 2011. Genomics for the world. Nature 475, 163–165. 21753830 10.1038/475163a PMC3708540

[R3] Chang M, Womer FY, Edmiston EK, Bai C, Zhou Q, Jiang X, Wei S, Wei Y, Ye Y, Huang H, He Y, Xu K, Tang Y, Wang F, 2018. Neurobiological commonalities and distinctions among three major psychiatric diagnostic categories: a structural MRI study. Schizophr. Bull 44, 65–74. 29036668 10.1093/schbul/sbx028 PMC5768040

[R4] Choudhury S, 2010. Culturing the adolescent brain: what can neuroscience learn from anthropology? Soc. Cogn. Affect. Neurosci 5, 159–167. 19959484 10.1093/scan/nsp030 PMC2894667

[R5] Foulkes L, Blakemore SJ, 2018. Studying individual differences in human adolescent brain development. Nat. Neurosci 21, 315–323. 29403031 10.1038/s41593-018-0078-4

[R6] Freeman A, Tyrovolas S, Koyanagi A, Chatterji S, Leonardi M, Ayuso-Mateos JL, Tobiasz-Adamczyk B, Koskinen S, Rummel-Kluge C, Haro JM, 2016. The role of socio-economic status in depression: results from the COURAGE (aging survey in Europe). BMC Public Health 16, 1098. 27760538 10.1186/s12889-016-3638-0 PMC5069819

[R7] Goddings AL, Mills KL, Clasen LS, Giedd JN, Viner RM, Blakemore SJ, 2014. The influence of puberty on subcortical brain development. Neuroimage 88, 242–251. 24121203 10.1016/j.neuroimage.2013.09.073 PMC3991320

[R8] Gong B, Naveed S, Hafeez DM, Afzal KI, Majeed S, Abele J, Nicolaou S, Khosa F, 2019. Neuroimaging in psychiatric disorders: a bibliometric analysis of the 100 most highly cited articles. J. Neuroimaging 29, 14–33. 30311320 10.1111/jon.12570

[R9] Han S, Ma Y, 2014. Cultural differences in human brain activity: a quantitative meta-analysis. Neuroimage 99, 293–300. 24882220 10.1016/j.neuroimage.2014.05.062

[R10] Hindorff LA, Bonham VL, Brody LC, Ginoza MEC, Hutter CM, Manolio TA, Green ED, 2018. Prioritizing diversity in human genomics research. Nat. Rev. Genet 19, 175–185. 29151588 10.1038/nrg.2017.89 PMC6532668

[R11] Hogea LM, Nussbaum LA, Chiriac DV, Ageu LS, Andreescu NI, Grigoras ML, Folescu R, Bredicean AC, Puiu M, Rosca ECI, Simu MA, Levai CM, 2017. Integrative clinico-biological, pharmacogenetic, neuroimagistic, neuroendocrinological and psychological correlations in depressive and anxiety disorders. Rom. J. Morphol. Embryol 58, 767–775. 29250653

[R12] Kerestes R, Davey CG, Stephanou K, Whittle S, Harrison BJ, 2014. Functional brain imaging studies of youth depression: a systematic review. Neuroimage Clin. 4, 209–231. 24455472 10.1016/j.nicl.2013.11.009 PMC3895619

[R13] Kieling C, Baker-Henningham H, Belfer M, Conti G, Ertem I, Omigbodun O, Rohde LA, Srinath S, Ulkuer N, Rahman A, 2011. Child and adolescent mental health worldwide: evidence for action. Lancet 378, 1515–1525. 22008427 10.1016/S0140-6736(11)60827-1

[R14] Kieling C, Rohde LA, 2012. Child and adolescent mental health research across the globe. J. Am. Acad. Child Adolesc. Psychiatry 51, 945–947. 22917207 10.1016/j.jaac.2012.07.002

[R15] McLane HC, Berkowitz AL, Patenaude BN, McKenzie ED, Wolper E, Wahlster S, Fink G, Mateen FJ, 2015. Availability, accessibility, and affordability of neurodiagnostic tests in 37 countries. Neurology 85, 1614–1622. 26446063 10.1212/WNL.0000000000002090 PMC4642148

[R16] Pan PM, Sato JR, Salum GA, Rohde LA, Gadelha A, Zugman A, Mari J, Jackowski A, Picon F, Miguel EC, Pine DS, Leibenluft E, Bressan RA, Stringaris A, 2017. Ventral striatum functional connectivity as a predictor of adolescent depressive disorder in a longitudinal community-based sample. Am. J. Psychiatry 174, 1112–1119. 28946760 10.1176/appi.ajp.2017.17040430 PMC10752293

[R17] Popejoy AB, Fullerton SM, 2016. Genomics is failing on diversity. Nature 538, 161–164. 27734877 10.1038/538161a PMC5089703

[R18] Sawyer SM, Azzopardi PS, Wickremarathne D, Patton GC, 2018. The age of adolescence. Lancet Child Adolesc. Health 2, 223–228. 30169257 10.1016/S2352-4642(18)30022-1

[R19] Stein DJ, Wegener G, 2017. Discovery versus implementation research on mental disorders in low- and middle-income countries. Acta Neuropsychiatr. 29, 191–192. 28793939 10.1017/neu.2017.25

[R20] Thapar A, Collishaw S, Pine DS, Thapar AK, 2012. Depression in adolescence. Lancet 379, 1056–1067. 22305766 10.1016/S0140-6736(11)60871-4 PMC3488279

[R21] World Bank. GNI, Atlas method (current US$), 2018a. https://data.worldbank.org/indicator/NY.GNP.ATLS.CD (Accessed 10 January 2020).

[R22] World Bank. Population, total, 2018b. https://data.worldbank.org/indicator/SP.POP.TOTL (Accessed 10 January 2020).

[R23] World Bank. World bank country and lending groups, 2020. https://datahelpdesk.worldbank.org/knowledgebase/articles/906519 (Accessed 10 January 2020).

[R24] Worthman C, Trang K, 2018. Dynamics of body time, social time and life history at adolescence. Nature 554, 451–457. 29469099 10.1038/nature25750

[R25] Zucker RA, Gonzalez R, Feldstein Ewing SW, Paulus MP, Arroyo J, Fuligni A, Morris AS, Sanchez M, Wills T, 2018. Assessment of culture and environment in the adolescent brain and cognitive development study: rationale, description of measures, and early data. Dev. Cogn. Neurosci 32, 107–120. 29627333 10.1016/j.dcn.2018.03.004 PMC6436615

