# Supplementary material for: Mind the brain gap: The worldwide distribution of neuroimaging research on adolescent depression
Source: Neuroimage. Author manuscript; Available in PMC 2022 May 1. (PMC8328473; doi:10.1016/j.neuroimage.2021.117865)
Supplement: Supplementary1 [file NIHMS1728112-supplement-Supplementary1.docx]

**Supplementary Material 1**. Full search syntax.

MEDLINE

((adolescent[mh] OR adolesc*[tw] OR young adult*[tw] OR youth[tw] OR teen*[tw] OR young people[tw] OR young person*[tw] OR boy[tw] OR boys[tw] OR girl[tw] OR girls[tw] OR pubescen*[tw] OR student*[tw] OR school[tw] OR child[mh:noexp] OR child[tw] OR children[tw] OR childhood[tw]) AND (mood disorders[mh] OR mood disorder*[tw] OR depression[tw] OR depressive[tw] OR MDD[tw] OR anhedonia[mh] OR anhedoni*[tw] OR dysthymi*[tw]) AND (neuroimaging[mh:noexp] OR functional neuroimaging[mh] OR neuroimag*[tw] OR functional neuroimag*[tw] OR brain imag*[tw] OR neurobiology[mh] OR neurobiolog*[tw] OR MRI[tw] OR fMRI[tw] OR magnetic resonance imaging[mh] OR magnetic resonance imag*[tw] OR resting state functional connect*[tw] OR diffusion tensor imaging[mh] OR diffusion tensor imag*[tw] OR DTI[tw] OR magnetic resonance spectroscop*[tw] OR MRS))

Web of Science (all databases)

((ALL=(adolesc* OR young adult* OR youth OR teen* OR young people OR young person* OR boy* OR girl* OR pubescen* OR student* OR school OR child*)) AND (ALL=(mood disorder* OR depressi* OR MDD OR anhedon* OR dysthymi*)) AND (ALL=(neuroimag* OR brain imag* OR neurobiolog* OR MRI OR fMRI OR magnetic resonance imaging OR resting-state functional connectivity OR diffusion tensor imaging OR DTI OR magnetic resonance spectroscopy OR MRS)))
