# Supplementary material for: Mind the brain gap: The worldwide distribution of neuroimaging research on adolescent depression
Source: Neuroimage. Author manuscript; Available in PMC 2022 May 1. (PMC8328473; doi:10.1016/j.neuroimage.2021.117865)
Supplement: Supplementary2 [file NIHMS1728112-supplement-Supplementary2.docx]

**Supplementary Material 2**. PRISMA flow diagram.

Duplicates excluded
(n = 4,186)

Records identified through database searching
(n = 10,659)

Records excluded
(n = 5,937)

Records screened
(n = 6,473)

Full-text articles excluded
(n = 388)

273 mean age >24

64 no depressed group

21 bipolar depression

16 no neuroimaging

10 mean age <10

4 no original data

Full-text articles assessed for eligibility
(n = 536)

Studies included in qualitative synthesis
(n = 148)
