# Supplementary material for: Mind the brain gap: The worldwide distribution of neuroimaging research on adolescent depression
Source: Neuroimage. Author manuscript; Available in PMC 2022 May 1. (PMC8328473; doi:10.1016/j.neuroimage.2021.117865)
Supplement: Supplementary3 [file NIHMS1728112-supplement-Supplementary3.docx]

**Supplementary Material 3.** Full reference list of all included studies.

Aghajani, M., Veer, I.M., van Lang, N.D., Meens, P.H., van den Bulk, B.G., Rombouts, S.A., Vermeiren, R.R., van der Wee, N.J., 2014. Altered white-matter architecture in treatment-naive adolescents with clinical depression. Psychological Medicine 44, 2287-2298.

Anand, A., Jones, S.E., Lowe, M., Karne, H., Koirala, P., 2018. Resting state functional connectivity of dorsal raphe nucleus and ventral tegmental area in medication-free young adults with major depression. Frontiers in Psychiatry 9, 765.

Battel, L., Swartz, J., Anes, M., Manfro, P.H., Rohde, L.A., Viduani, A., Mondelli, V., Kieling, C., 2019. Neuroimaging adolescents with depression in a middle-income country: feasibility of an fMRI protocol and preliminary results. Braz J Psychiatry, S1516-44462019005013104.

Beesdo, K., Lau, J.Y., Guyer, A.E., McClure-Tone, E.B., Monk, C.S., Nelson, E.E., Fromm, S.J., Goldwin, M.A., Wittchen, H.U., Leibenluft, E., Ernst, M., Pine, D.S., 2009. Common and distinct amygdala-function perturbations in depressed vs anxious adolescents. Archives of General Psychiatry 66, 275-285.

Bessette, K.L., Jenkins, L.M., Skerrett, K.A., Gowins, J.R., DelDonno, S.R., Zubieta, J.K., McInnis, M.G., Jacobs, R.H., Ajilore, O., Langenecker, S.A., 2018. Reliability, convergent validity and time invariance of default mode network deviations in early adult major depressive disorder. Frontiers in Psychiatry 9, 244.

Bos, M.G.N., Peters, S., van de Kamp, F.C., Crone, E.A., Tamnes, C.K., 2018. Emerging depression in adolescence coincides with accelerated frontal cortical thinning. Journal of Child Psychology and Psychiatry 59, 994-1002.

Botteron, K.N., Raichle, M.E., Drevets, W.C., Heath, A.C., Todd, R.D., 2002. Volumetric reduction in left subgenual prefrontal cortex in early onset depression. Biological Psychiatry 51, 342-344.

Burkhouse, K.L., Jacobs, R.H., Peters, A.T., Ajilore, O., Watkins, E.R., Langenecker, S.A., 2017. Neural correlates of rumination in adolescents with remitted major depressive disorder and healthy controls. Cognitive, Affective & Behavioral Neuroscience 17, 394-405.

Caetano, S.C., Fonseca, M., Hatch, J.P., Olvera, R.L., Nicoletti, M., Hunter, K., Lafer, B., Pliszka, S.R., Soares, J.C., 2007. Medial temporal lobe abnormalities in pediatric unipolar depression. Neuroscience Letters 427, 142-147.

Caetano, S.C., Fonseca, M., Olvera, R.L., Nicoletti, M., Hatch, J.P., Stanley, J.A., Hunter, K., Lafer, B., Pliszka, S.R., Soares, J.C., 2005. Proton spectroscopy study of the left dorsolateral prefrontal cortex in pediatric depressed patients. Neuroscience Letters 384, 321-326.

Callaghan, B.L., Dandash, O., Simmons, J.G., Schwartz, O., Byrne, M.L., Sheeber, L., Allen, N.B., Whittle, S., 2017. Amygdala resting connectivity mediates association between maternal aggression and adolescent major depression: a 7-year longitudinal Sstudy. Journal of the American Academy of Child and Adolescent Psychiatry 56, 983-991.e983.

Cao, J., Ai, M., Chen, X., Chen, J., Wang, W., Kuang, L., 2020. Altered resting-state functional network connectivity is associated with suicide attempt in young depressed patients. Psychiatry Res 285, 112713.

Cao, J., Chen, X., Chen, J., Ai, M., Gan, Y., Wang, W., Lv, Z., Zhang, S., Zhang, S., Wang, S., Kuang, L., Fang, W., 2016. Resting-state functional MRI of abnormal baseline brain activity in young depressed patients with and without suicidal behavior. Journal of Affective Disorders 205, 252-263.

Carew, C.L., Tatham, E.L., Milne, A.M., MacQueen, G.M., Hall, G.B., 2015. Design and implementation of an fMRI study examining thought suppression in young women with, and at-risk, for depression. Journal of Visualized Experiments : JoVE, e52061.

Carpenter, J.S., Abelmann, A.C., Hatton, S.N., Robillard, R., Hermens, D.F., Bennett, M.R., Lagopoulos, J., Hickie, I.B., 2017. Pineal volume and evening melatonin in young people with affective disorders. Brain Imaging and Behavior 11, 1741-1750.

Chahal, R., Weissman, D.G., Marek, S., Rhoads, S.A., Hipwell, A.E., Forbes, E.E., Keenan, K., Guyer, A.E., 2019. Girls' brain structural connectivity in late adolescence relates to history of depression symptoms. Journal of Child Psychology and Psychiatry, 10.1111/jcpp.13184.

Chan, S.W., Sussmann, J.E., Romaniuk, L., Stewart, T., Lawrie, S.M., Hall, J., McIntosh, A.M., Whalley, H.C., 2016. Deactivation in anterior cingulate cortex during facial processing in young individuals with high familial risk and early development of depression: fMRI findings from the Scottish Bipolar Family Study. Journal of child psychology and psychiatry, and allied disciplines 57, 1277-1286.

Chang, M., Womer, F.Y., Edmiston, E.K., Bai, C., Zhou, Q., Jiang, X., Wei, S., Wei, Y., Ye, Y., Huang, H., He, Y., Xu, K., Tang, Y., Wang, F., 2018. Neurobiological commonalities and distinctions among three major psychiatric diagnostic categories: a structural MRI study. Schizophrenia bulletin 44, 65-74.

Chen, H.H., Rosenberg, D.R., MacMaster, F.P., Easter, P.C., Caetano, S.C., Nicoletti, M., Hatch, J.P., Nery, F.G., Soares, J.C., 2008. Orbitofrontal cortex volumes in medication naive children with major depressive disorder: a magnetic resonance imaging study. Journal of child and adolescent psychopharmacology 18, 551-556.

Chen, J.D., Liu, F., Xun, G.L., Chen, H.F., Hu, M.R., Guo, X.F., Xiao, C.Q., Wooderson, S.C., Guo, W.B., Zhao, J.P., 2012. Early and late onset, first-episode, treatment-naive depression: same clinical symptoms, different regional neural activities. Journal of affective disorders 143, 56-63.

Chu, S.H., Lenglet, C., Schreiner, M.W., Klimes-Dougan, B., Cullen, K., Parhi, K.K., 2018. Anatomical biomarkers for adolescent major depressive disorder from diffusion weighted imaging using SVM classifier. Annu Int Conf IEEE Eng Med Biol Soc 2018, 2740-2743.

Chu, S.H., Lenglet, C., Schreiner, M.W., Klimes-Dougan, B., Cullen, K., Parhi, K.K., 2018. Classifying Treated vs. Untreated MDD Adolescents from Anatomical Connectivity using Nonlinear SVM. Annu Int Conf IEEE Eng Med Biol Soc 2018, 1-4.

Cornelius, J.R., Aizenstein, H.J., Chung, T.A., Douaihy, A., Hayes, J., Daley, D., Salloum, I.M., 2013. Paradoxical decrease in striatal activation on an fMRI reward task following treatment in youth with co-morbid cannabis dependence/major depression. Advances in psychology research 93, 123-130.

Cornelius, J.R., Aizenstein, H.J., Hariri, A.R., 2010. Amygdala reactivity is inversely related to level of cannabis use in individuals with comorbid cannabis dependence and major depression. Addictive behaviors 35, 644-646.

Cullen, K.R., Brown, R., Schreiner, M.W., Eberly, L.E., Klimes-Dougan, B., Reigstad, K., Hill, D., Lim, K.O., Mueller, B.A., 2019. White matter microstructure relates to lassitude but not diagnosis in adolescents with depression. Brain imaging and behavior.

Davey, C.G., Breakspear, M., Pujol, J., Harrison, B.J., 2017. A brain model of disturbed self-appraisal in depression. The American journal of psychiatry 174, 895-903.

Davey, C.G., Harrison, B.J., Yucel, M., Allen, N.B., 2012a. Regionally specific alterations in functional connectivity of the anterior cingulate cortex in major depressive disorder. Psychological medicine 42, 2071-2081.

Davey, C.G., Yucel, M., Allen, N.B., Harrison, B.J., 2012b. Task-related deactivation and functional connectivity of the subgenual cingulate cortex in major depressive disorder. Frontiers in psychiatry 3, 14.

De Bellis, M.D., Hooper, S.R., 2012. Neural substrates for processing task-irrelevant emotional distracters in maltreated adolescents with depressive disorders: a pilot study. Journal of traumatic stress 25, 198-202.

De Somma, E., Jaworska, N., Courtright, A., Bray, S., Lebel, R.M., MacMaster, F., MacQueen, G.M., 2018. A pilot study of hippocampal activity during a verbal memory task in depressed young adults. Adolescent Psychiatry 8, 21-31.

Dichter, G.S., Kozink, R.V., McClernon, F.J., Smoski, M.J., 2012. Remitted major depression is characterized by reward network hyperactivation during reward anticipation and hypoactivation during reward outcomes. Journal of affective disorders 136, 1126-1134.

Diler, R.S., de Almeida, J.R., Ladouceur, C., Birmaher, B., Axelson, D., Phillips, M., 2013. Neural activity to intense positive versus negative stimuli can help differentiate bipolar disorder from unipolar major depressive disorder in depressed adolescents: a pilot fMRI study. Psychiatry research 214, 277-284.

Diler, R.S., Pan, L.A., Segreti, A., Ladouceur, C.D., Forbes, E., Cela, S.R., Almeida, J.R., Birmaher, B., Axelson, D.A., Phillips, M.L., 2014. Differential anterior cingulate activity during response inhibition in depressed adolescents with bipolar and unipolar major depressive disorder. Journal of the Canadian Academy of Child and Adolescent Psychiatry = Journal de l'Academie canadienne de psychiatrie de l'enfant et de l'adolescent 23, 10-19.

Dong, D., Ming, Q., Zhong, X., Pu, W., Zhang, X., Jiang, Y., Gao, Y., Sun, X., Wang, X., Yao, S., 2019. State-independent alterations of intrinsic brain network in current and remitted depression. Progress in neuro-psychopharmacology & biological psychiatry 89, 475-480.

Ehrlich, S., Noam, G.G., Lyoo, I.K., Kwon, B.J., Clark, M.A., Renshaw, P.F., 2004. White matter hyperintensities and their associations with suicidality in psychiatrically hospitalized children and adolescents. Journal of the American Academy of Child and Adolescent Psychiatry 43, 770-776.

Fallucca, E., MacMaster, F.P., Haddad, J., Easter, P., Dick, R., May, G., Stanley, J.A., Rix, C., Rosenberg, D.R., 2011. Distinguishing between major depressive disorder and obsessive-compulsive disorder in children by measuring regional cortical thickness. Archives of general psychiatry 68, 527-533.

Fischer, A.S., Ellwood-Lowe, M.E., Colich, N.L., Cichocki, A., Ho, T.C., Gotlib, I.H., 2019. Reward-circuit biomarkers of risk and resilience in adolescent depression. Journal of affective disorders 246, 902-909.

Forbes, E.E., Christopher May, J., Siegle, G.J., Ladouceur, C.D., Ryan, N.D., Carter, C.S., Birmaher, B., Axelson, D.A., Dahl, R.E., 2006. Reward-related decision-making in pediatric major depressive disorder: an fMRI study. Journal of child psychology and psychiatry, and allied disciplines 47, 1031-1040.

Forbes, E.E., Hariri, A.R., Martin, S.L., Silk, J.S., Moyles, D.L., Fisher, P.M., Brown, S.M., Ryan, N.D., Birmaher, B., Axelson, D.A., Dahl, R.E., 2009. Altered striatal activation predicting real-world positive affect in adolescent major depressive disorder. The American journal of psychiatry 166, 64-73.

Ford, K.A., Theberge, J., Neufeld, R.J., Williamson, P.C., Osuch, E.A., 2013. Correlation of brain default mode network activation with bipolarity index in youth with mood disorders. Journal of affective disorders 150, 1174-1178.

Ford, K.A., Wammes, M., Neufeld, R.W., Mitchell, D., Theberge, J., Williamson, P., Osuch, E.A., 2014. Unique functional abnormalities in youth with combined marijuana use and depression: an FMRI study. Frontiers in psychiatry 5, 130.

Gong, Y., Hao, L., Zhang, X., Zhou, Y., Li, J., Zhao, Z., Jiang, W., Du, Y., 2014. Case-control resting-state fMRI study of brain functioning among adolescents with first-episode major depressive disorder. Shanghai Arch Psychiatry 26, 207-215.

Griffiths, K.R., Lagopoulos, J., Hermens, D.F., Hickie, I.B., Balleine, B.W., 2015. Right external globus pallidus changes are associated with altered causal awareness in youth with depression. Translational psychiatry 5, e653.

Gruskin, D.C., Rosenberg, M.D., Holmes, A.J., 2019. Relationships between depressive symptoms and brain responses during emotional movie viewing emerge in adolescence. NeuroImage, 116217-116217.

Henderson, S.E., Johnson, A.R., Vallejo, A.I., Katz, L., Wong, E., Gabbay, V., 2013. A preliminary study of white matter in adolescent depression: relationships with illness severity, anhedonia, and irritability. Front Psychiatry 4, 152.

Hermens, D.F., Chitty, K.M., Lee, R.S., Tickell, A., Haber, P.S., Naismith, S.L., Hickie, I.B., Lagopoulos, J., 2015. Hippocampal glutamate is increased and associated with risky drinking in young adults with major depression. Journal of affective disorders 186, 95-98.

Ho, T.C., Sacchet, M.D., Connolly, C.G., Margulies, D.S., Tymofiyeva, O., Paulus, M.P., Simmons, A.N., Gotlib, I.H., Yang, T.T., 2017. Inflexible functional connectivity of the dorsal anterior cingulate cortex in adolescent major depressive disorder. Neuropsychopharmacology: official publication of the American College of Neuropsychopharmacology 42, 2434-2445.

Hogea, L.M., Nussbaum, L.A., Chiriac, D.V., Ageu, L.S., Andreescu, N.I., Grigoras, M.L., Folescu, R., Bredicean, A.C., Puiu, M., Rosca, E.C.I., Simu, M.A., Levai, C.M., 2017. Integrative clinico-biological, pharmacogenetic, neuroimagistic, neuroendocrinological and psychological correlations in depressive and anxiety disorders. Romanian journal of morphology and embryology = Revue roumaine de morphologie et embryologie 58, 767-775.

Hu, L., Xiao, M., Ai, M., Wang, W., Chen, J., Tan, Z., Cao, J., Kuang, L., 2019. Disruption of resting-state functional connectivity of right posterior insula in adolescents and young adults with major depressive

disorder. Journal of affective disorders 257, 23-30.

Jacobs, R.H., Jenkins, L.M., Gabriel, L.B., Barba, A., Ryan, K.A., Weisenbach, S.L., Verges, A., Baker, A.M., Peters, A.T., Crane, N.A., Gotlib, I.H., Zubieta, J.K., Phan, K.L., Langenecker, S.A., Welsh, R.C., 2014. Increased coupling of intrinsic networks in remitted depressed youth predicts rumination and cognitive control. PloS one 9, e104366.

Jaworska, N., Yucel, K., Courtright, A., MacMaster, F.P., Sembo, M., MacQueen, G., 2016. Subgenual anterior cingulate cortex and hippocampal volumes in depressed youth: The role of comorbidity and age. Journal of affective disorders 190, 726-732.

Jenkins, L.M., Kassel, M.T., Gabriel, L.B., Gowins, J.R., Hymen, E.A., Verges, A., Calamia, M., Crane, N.A., Jacobs, R.H., Ajilore, O., Welsh, R.C., Drevets, W.C., Phillips, M.L., Zubieta, J.K., Langenecker, S.A., 2016. Amygdala and dorsomedial hyperactivity to emotional faces in youth with remitted Major Depression. Social cognitive and affective neuroscience 11, 736-745.

Jenkins, L.M., Kendall, A.D., Kassel, M.T., Patron, V.G., Gowins, J.R., Dion, C., Shankman, S.A., Weisenbach, S.L., Maki, P., Langenecker, S.A., 2018a. Considering sex differences clarifies the effects of depression on facial emotion processing during fMRI. Journal of affective disorders 225, 129-136.

Jenkins, L.M., Stange, J.P., Bessette, K.L., Chang, Y.S., Corwin, S.D., Skerrett, K.A., Patron, V.G., Zubieta, J.K., Crane, N.A., Passarotti, A.M., Pine, D.S., Langenecker, S.A., 2018b. Differential engagement of cognitive control regions and subgenual cingulate based upon presence or absence of comorbid anxiety with depression. Journal of affective disorders 241, 371-380.

Jiang, X., Dai, X., Kale Edmiston, E., Zhou, Q., Xu, K., Zhou, Y., Wu, F., Kong, L., Wei, S., Zhou, Y., Chang, M., Geng, H., Wang, D., Wang, Y., Cui, W., Wang, F., Tang, Y., 2017. Alteration of cortico-limbic-striatal neural system in major depressive disorder and bipolar disorder. Journal of affective disorders 221, 297-303.

Kaiser, R.H., Kang, M.S., Lew, Y., Van Der Feen, J., Aguirre, B., Clegg, R., Goer, F., Esposito, E., Auerbach, R.P., Hutchison, R.M., Pizzagalli, D.A., 2019. Abnormal frontoinsular-default network dynamics in adolescent depression and rumination: a preliminary resting-state co-activation pattern analysis. Neuropsychopharmacology : official publication of the American College of Neuropsychopharmacology.

Kerestes, R., Harrison, B.J., Dandash, O., Stephanou, K., Whittle, S., Pujol, J., Davey, C.G., 2015. Specific functional connectivity alterations of the dorsal striatum in young people with depression. NeuroImage. Clinical 7, 266-272.

Kim, J.H., Joo, Y.H., Son, Y.D., Kim, J.H., Kim, Y.K., Kim, H.K., Lee, S.Y., Ido, T., 2019a. In vivo metabotropic glutamate receptor 5 availability-associated functional connectivity alterations in drug-naive young adults with major depression. European neuropsychopharmacology : the journal of the European College of Neuropsychopharmacology 29, 278-290.

Kim, J.H., Suh, S.-I., Lee, H.-J., Lee, J.-H., Lee, M.-S., 2019b. Cortical and subcortical gray matter alterations in first-episode drug-naïve adolescents with major depressive disorder. Neuroreport 30, 1172-1178.

Koenig, J., Westlund Schreiner, M., Klimes-Dougan, B., Ubani, B., Mueller, B.A., Lim, K.O., Kaess, M., Cullen, K.R., 2018. Increases in orbitofrontal cortex thickness following antidepressant treatment are associated with changes in resting state autonomic function in adolescents with major depression - Preliminary findings from a pilot study. Psychiatry research. Neuroimaging 281, 35-42.

Kondo, D.G., Forrest, L.N., Shi, X., Sung, Y.H., Hellem, T.L., Huber, R.S., Renshaw, P.F., 2016. Creatine target engagement with brain bioenergetics: a dose-ranging phosphorus-31 magnetic resonance spectroscopy study of adolescent females with SSRI-resistant depression. Amino acids 48, 1941-1954.

Kondo, D.G., Sung, Y.H., Hellem, T.L., Fiedler, K.K., Shi, X., Jeong, E.K., Renshaw, P.F., 2011. Open-label adjunctive creatine for female adolescents with SSRI-resistant major depressive disorder: a 31-phosphorus magnetic resonance spectroscopy study. Journal of affective disorders 135, 354-361.

Langenecker, S.A., Jenkins, L.M., Stange, J.P., Chang, Y.S., DelDonno, S.R., Bessette, K.L., Passarotti, A.M., Bhaumik, R., Ajilore, O., Jacobs, R.H., 2018. Cognitive control neuroimaging measures differentiate between those with and without future recurrence of depression. NeuroImage. Clinical 20, 1001-1009.

Lau, J.Y., Goldman, D., Buzas, B., Hodgkinson, C., Leibenluft, E., Nelson, E., Sankin, L., Pine, D.S., Ernst, M., 2010. BDNF gene polymorphism (Val66Met) predicts amygdala and anterior hippocampus responses to emotional faces in anxious and depressed adolescents. NeuroImage 53, 952-961.

Le, T.M., Borghi, J.A., Kujawa, A.J., Klein, D.N., Leung, H.C., 2017. Alterations in visual cortical activation and connectivity with prefrontal cortex during working memory updating in major depressive disorder. NeuroImage. Clinical 14, 43-53.

Lee, D., Lee, J., Namkoong, K., Jung, Y.C., 2018. Subregions of the anterior cingulate cortex form distinct functional connectivity patterns in young males with internet gaming disorder with comorbid depression. Frontiers in psychiatry 9, 380.

Lefebvre, D., Langevin, L.M., Jaworska, N., Harris, A.D., Lebel, R.M., Jasaui, Y., Kirton, A., Wilkes, T.C., Sembo, M., Swansburg, R., MacMaster, F.P., 2017. A pilot study of hippocampal N-acetyl-aspartate in youth with treatment resistant major depression. Journal of affective disorders 207, 110-113.

Leming, M., Su, L., Chattopadhyay, S., Suckling, J., 2019. Normative pathways in the functional connectome. NeuroImage 184, 317-334.

Lewis, C.P., Port, J.D., Frye, M.A., Vande Voort, J.L., Ameis, S.H., Husain, M.M., Daskalakis, Z.J., Croarkin, P.E., 2016. An exploratory study of spectroscopic glutamatergic correlates of cortical excitability in depressed adolescents. Frontiers in neural circuits 10, 98.

Lippard, E.T.C., Johnston, J.A.Y., Spencer, L., Quatrano, S., Fan, S., Sankar, A., Weathers, J., Pittman, B., Oquendo, M.A., Blumberg, H.P., 2019. Preliminary examination of gray and white matter structure and longitudinal structural changes in frontal systems associated with future suicide attempts in adolescents and young adults with mood disorders. Journal of affective disorders 245, 1139-1148.

Lopez, K.C., Luby, J.L., Belden, A.C., Barch, D.M., 2018. Emotion dysregulation and functional connectivity in children with and without a history of major depressive disorder. Cognitive, affective & behavioral neuroscience 18, 232-248.

Luby, J.L., Agrawal, A., Belden, A., Whalen, D., Tillman, R., Barch, D.M., 2018. Developmental trajectories of the orbitofrontal cortex and anhedonia in middle childhood and risk for substance use in adolescence in a longitudinal sample of depressed and healthy preschoolers. The American journal of psychiatry 175, 1010-1021.

Lyoo, I.K., Kwon, J.S., Lee, S.J., Han, M.H., Chang, C.G., Seo, C.S., Lee, S.I., Renshaw, P.F., 2002. Decrease in genu of the corpus callosum in medication-naive, early-onset dysthymia and depressive personality disorder. Biological psychiatry 52, 1134-1143.

MacMaster, F.P., Mirza, Y., Szeszko, P.R., Kmiecik, L.E., Easter, P.C., Taormina, S.P., Lynch, M., Rose, M., Moore, G.J., Rosenberg, D.R., 2008a. Amygdala and hippocampal volumes in familial early onset major depressive disorder. Biological psychiatry 63, 385-390.

MacMaster, F.P., Moore, G.J., Russell, A., Mirza, Y., Taormina, S.P., Buhagiar, C., Rosenberg, D.R., 2008b. Medial temporal N-acetyl-aspartate in pediatric major depression. Psychiatry research 164, 86-89.

MacMaster, F.P., Russell, A., Mirza, Y., Keshavan, M.S., Taormina, S.P., Bhandari, R., Boyd, C., Lynch, M., Rose, M., Ivey, J., Moore, G.J., Rosenberg, D.R., 2006. Pituitary volume in treatment-naive pediatric major depressive disorder. Biological psychiatry 60, 862-866.

Matthews, S.C., Strigo, I.A., Simmons, A.N., Yang, T.T., Paulus, M.P., 2008. Decreased functional coupling of the amygdala and supragenual cingulate is related to increased depression in unmedicated individuals with current major depressive disorder. J Affect Disord 111, 13-20.

McLellan, Q., Wilkes, T.C., Swansburg, R., Jaworska, N., Langevin, L.M., MacMaster, F.P., 2018. History of suicide attempt and right superior temporal gyrus volume in youth with treatment-resistant major depressive disorder. Journal of affective disorders 239, 291-294.

McNamara, R.K., Jandacek, R., Rider, T., Tso, P., Chu, W.J., Weber, W.A., Welge, J.A., Strawn, J.R., Adler, C.M., DelBello, M.P., 2016. Effects of fish oil supplementation on prefrontal metabolite concentrations in adolescents with major depressive disorder: a preliminary 1H MRS study. Nutritional neuroscience 19, 145-155.

Ming, Q., Zhong, X., Zhang, X., Pu, W., Dong, D., Jiang, Y., Gao, Y., Wang, X., Detre, J.A., Yao, S., Rao, H., 2017. State-independent and dependent neural responses to psychosocial stress in current and remitted depression. The American journal of psychiatry 174, 971-979.

Mirza, Y., O'Neill, J., Smith, E.A., Russell, A., Smith, J.M., Banerjee, S.P., Bhandari, R., Boyd, C., Rose, M., Ivey, J., Renshaw, P.F., Rosenberg, D.R., 2006. Increased medial thalamic creatine-phosphocreatine found by proton magnetic resonance spectroscopy in children with obsessive-compulsive disorder versus major depression and healthy controls. Journal of child neurology 21, 106-111.

Morgan, J.K., Shaw, D.S., Olino, T.M., Musselman, S.C., Kurapati, N.T., Forbes, E.E., 2016. History of depression and frontostriatal connectivity during reward processing in late adolescent boys. Journal of clinical child and adolescent psychology : the official journal for the Society of Clinical Child and Adolescent Psychology, American Psychological Association, Division 53 45, 59-68.

Munn, M.A., Alexopoulos, J., Nishino, T., Babb, C.M., Flake, L.A., Singer, T., Ratnanather, J.T., Huang, H., Todd, R.D., Miller, M.I., Botteron, K.N., 2007. Amygdala volume analysis in female twins with major depression. Biological psychiatry 62, 415-422.

Murphy, E.R., Barch, D.M., Pagliaccio, D., Luby, J.L., Belden, A.C., 2016. Functional connectivity of the amygdala and subgenual cingulate during cognitive reappraisal of emotions in children with MDD history is associated with rumination. Developmental cognitive neuroscience 18, 89-100.

Nickson, T., Chan, S.W., Papmeyer, M., Romaniuk, L., Macdonald, A., Stewart, T., Kielty, S., Lawrie, S.M., Hall, J., Sussmann, J.E., McIntosh, A.M., Whalley, H.C., 2016. Prospective longitudinal voxel-based morphometry study of major depressive disorder in young individuals at high familial risk. Psychological medicine 46, 2351-2361.

Nimarko, A.F., Garrett, A.S., Carlson, G.A., Singh, M.K., 2019. Neural correlates of emotion processing predict resilience in youth at familial risk for mood disorders. Development and psychopathology, 1-16.

Nolan, C.L., Moore, G.J., Madden, R., Farchione, T., Bartoi, M., Lorch, E., Stewart, C.M., Rosenberg, D.R., 2002. Prefrontal cortical volume in childhood-onset major depression: preliminary findings. Archives of general psychiatry 59, 173-179.

Olino, T.M., McMakin, D.L., Dahl, R.E., Ryan, N.D., Silk, J.S., Birmaher, B., Axelson, D.A., Forbes, E.E., 2011. "I won, but I'm not getting my hopes up": depression moderates the relationship of outcomes and reward anticipation. Psychiatry research 194, 393-395.

Olvera, R.L., Caetano, S.C., Stanley, J.A., Chen, H.H., Nicoletti, M., Hatch, J.P., Fonseca, M., Pliszka, S.R., Soares, J.C., 2010. Reduced medial prefrontal N-acetyl-aspartate levels in pediatric major depressive disorder: a multi-voxel in vivo(1)H spectroscopy study. Psychiatry research 184, 71-76.

Osuch, E.A., Manning, K., Hegele, R.A., Theberge, J., Neufeld, R., Mitchell, D., Williamson, P., Gardner, R.C., 2016. Depression, marijuana use and early-onset marijuana use conferred unique effects on neural connectivity and cognition. Acta psychiatrica Scandinavica 134, 399-409.

Pan, F., Xu, Y., Zhou, W., Chen, J., Wei, N., Lu, S., Shang, D., Wang, J., Huang, M., 2019. Disrupted intrinsic functional connectivity of the cognitive control network underlies disease severity and executive dysfunction in first-episode, treatment-naive adolescent depression. Journal of affective disorders, S0165-0327(0119)31839-31837.

Pan, L., Segreti, A., Almeida, J., Jollant, F., Lawrence, N., Brent, D., Phillips, M., 2013. Preserved hippocampal function during learning in the context of risk in adolescent suicide attempt. Psychiatry research 211, 112-118.

Pan, L.A., Batezati-Alves, S.C., Almeida, J.R., Segreti, A., Akkal, D., Hassel, S., Lakdawala, S., Brent, D.A., Phillips, M.L., 2011. Dissociable patterns of neural activity during response inhibition in depressed adolescents with and without suicidal behavior. Journal of the American Academy of Child and Adolescent Psychiatry 50, 602-611.e603.

Pan, L.A., Ramos, L., Segreti, A., Brent, D.A., Phillips, M.L., 2015. Right superior temporal gyrus volume in adolescents with a history of suicide attempt. The British journal of psychiatry : the journal of mental science 206, 339-340.

Pan, P.M., Sato, J.R., Salum, G.A., Rohde, L.A., Gadelha, A., Zugman, A., Mari, J., Jackowski, A., Picon, F., Miguel, E.C., Pine, D.S., Leibenluft, E., Bressan, R.A., Stringaris, A., 2017. Ventral striatum functional connectivity as a predictor of adolescent depressive disorder in a longitudinal community-based Sample. The American journal of psychiatry 174, 1112-1119.

Pannekoek, J.N., van der Werff, S.J., Meens, P.H., van den Bulk, B.G., Jolles, D.D., Veer, I.M., van Lang, N.D., Rombouts, S.A., van der Wee, N.J., Vermeiren, R.R., 2014. Aberrant resting-state functional connectivity in limbic and salience networks in treatment--naive clinically depressed adolescents. J Child Psychol Psychiatry 55, 1317-1327.

Penner, J., Osuch, E.A., Schaefer, B., Theberge, J., Neufeld, R.W.J., Menon, R.S., Rajakumar, N., Bourne, J.A., Williamson, P.C., 2018. Higher order thalamic nuclei resting network connectivity in early schizophrenia and major depressive disorder. Psychiatry research. Neuroimaging 272, 7-16.

Peters, A.T., Burkhouse, K., Feldhaus, C.C., Langenecker, S.A., Jacobs, R.H., 2016. Aberrant resting-state functional connectivity in limbic and cognitive control networks relates to depressive rumination and mindfulness: A pilot study among adolescents with a history of depression. Journal of affective disorders 200, 178-181.

Quevedo, K., Martin, J., Scott, H., Smyda, G., Pfeifer, J.H., 2016. The neurobiology of self-knowledge in depressed and self-injurious youth. Psychiatry research. Neuroimaging 254, 145-155.

Rao, J.A., Jenkins, L.M., Hymen, E., Feigon, M., Weisenbach, S.L., Zubieta, J.K., Langenecker, S.A., 2016. Differential resting state connectivity patterns and impaired semantically cued list learning test performance in early course remitted major depressive disorder. Journal of the International Neuropsychological Society : JINS 22, 225-239.

Rao, U., Chen, L.A., Bidesi, A.S., Shad, M.U., Thomas, M.A., Hammen, C.L., 2010. Hippocampal changes associated with early-life adversity and vulnerability to depression. Biological psychiatry 67, 357-364.

Redlich, R., Opel, N., Burger, C., Dohm, K., Grotegerd, D., Forster, K., Zaremba, D., Meinert, S., Repple, J., Enneking, V., Leehr, E., Bohnlein, J., Winters, L., Frobose, N., Thrun, S., Emtmann, J., Heindel, W., Kugel, H., Arolt, V., Romer, G., Postert, C., Dannlowski, U., 2018. The limbic system in youth depression: brain structural and functional alterations in adolescent in-patients with severe depression.

Neuropsychopharmacology: official publication of the American College of Neuropsychopharmacology 43, 546-554.

Riem, M.M.E., van Hoof, M.J., Garrett, A.S., Rombouts, S., van der Wee, N.J.A., van, I.M.H., Vermeiren, R., 2019. General psychopathology factor and unresolved-disorganized attachment uniquely correlated to white matter integrity using diffusion tensor imaging. Behavioural brain research 359, 1-8.

Rosenberg, D.R., Mirza, Y., Russell, A., Tang, J., Smith, J.M., Banerjee, S.P., Bhandari, R., Rose, M., Ivey, J., Boyd, C., Moore, G.J., 2004. Reduced anterior cingulate glutamatergic concentrations in childhood OCD and major depression versus healthy controls. Journal of the American Academy of Child and Adolescent Psychiatry 43, 1146-1153.

Schiller, C.E., Minkel, J., Smoski, M.J., Dichter, G.S., 2013. Remitted major depression is characterized by reduced prefrontal cortex reactivity to reward loss. Journal of affective disorders 151, 756-762.

Schmaal, L., Hibar, D.P., Samann, P.G., Hall, G.B., Baune, B.T., Jahanshad, N., Cheung, J.W., van Erp, T.G.M., Bos, D., Ikram, M.A., Vernooij, M.W., Niessen, W.J., Tiemeier, H., Hofman, A., Wittfeld, K., Grabe, H.J., Janowitz, D., Bulow, R., Selonke, M., Volzke, H., Grotegerd, D., Dannlowski, U., Arolt, V., Opel, N., Heindel, W., Kugel, H., Hoehn, D., Czisch, M., Couvy-Duchesne, B., Renteria, M.E., Strike, L.T., Wright, M.J., Mills, N.T., de Zubicaray, G.I., McMahon, K.L., Medland, S.E., Martin, N.G., Gillespie, N.A., Goya-Maldonado, R., Gruber, O., Kramer, B., Hatton, S.N., Lagopoulos, J., Hickie, I.B., Frodl, T., Carballedo, A., Frey, E.M., van Velzen, L.S., Penninx, B., van Tol, M.J., van der Wee, N.J., Davey, C.G., Harrison, B.J., Mwangi, B., Cao, B., Soares, J.C., Veer, I.M., Walter, H., Schoepf, D., Zurowski, B., Konrad, C., Schramm, E., Normann, C., Schnell, K., Sacchet, M.D., Gotlib, I.H., MacQueen, G.M., Godlewska, B.R., Nickson, T., McIntosh, A.M., Papmeyer, M., Whalley, H.C., Hall, J., Sussmann, J.E., Li, M., Walter, M., Aftanas, L., Brack, I., Bokhan, N.A., Thompson, P.M., Veltman, D.J., 2017. Cortical abnormalities in adults and adolescents with major depression based on brain scans from 20 cohorts worldwide in the ENIGMA Major Depressive Disorder Working Group. Molecular psychiatry 22, 900-909.

Schnyer, D.M., Clasen, P.C., Gonzalez, C., Beevers, C.G., 2017. Evaluating the diagnostic utility of applying a machine learning algorithm to diffusion tensor MRI measures in individuals with major depressive disorder. Psychiatry research. Neuroimaging 264, 1-9.

Schreiner, M.W., Klimes-Dougan, B., Cullen, K.R., 2018. Neural correlates of suicidality in adolescents with major depression: resting-state functional connectivity of the precuneus and posterior cingulate cortex. Suicide & life-threatening behavior.

Schwartz, K.T.G., Kryza-Lacombe, M., Liuzzi, M.T., Weersing, V.R., Wiggins, J.L., 2019. Social and non-social reward: a preliminary examination of clinical improvement and neural reactivity in adolescents treated with behavioral therapy for anxiety and depression. Front Behav Neurosci 13, 177-177.

Shad, M.U., Bidesi, A.P., Chen, L.A., Ernst, M., Rao, U., 2011. Neurobiology of decision making in depressed adolescents: a functional magnetic resonance imaging study. Journal of the American Academy of Child and Adolescent Psychiatry 50, 612-621.e612.

Shapero, B.G., Chai, X.J., Vangel, M., Biederman, J., Hoover, C.S., Whitfield-Gabrieli, S., Gabrieli, J.D.E., Hirshfeld-Becker, D.R., 2019. Neural markers of depression risk predict the onset of depression. Psychiatry research. Neuroimaging 285, 31-39.

Shi, H., Wang, X., Yi, J., Zhu, X., Zhang, X., Yang, J., Yao, S., 2015. Default mode network alterations during implicit emotional faces processing in first-episode, treatment-naive major depression patients. Frontiers in psychology 6, 1198.

Shiota, S., Okamoto, Y., Okada, G., Takagaki, K., Takamura, M., Mori, A., Yokoyama, S., Nishiyama, Y., Jinnin, R., Hashimoto, R.I., Yamawaki, S., 2017. Effects of behavioural activation on the neural basis of other perspective self-referential processing in subthreshold depression: a functional magnetic resonance imaging study. Psychological medicine 47, 877-888.

Singh, M.K., Leslie, S.M., Packer, M.M., Weisman, E.F., Gotlib, I.H., 2018. Limbic intrinsic connectivity in depressed and high-risk youth. Journal of the American Academy of Child and Adolescent Psychiatry 57, 775-785.e773.

Singh, M.K., Leslie, S.M., Packer, M.M., Zaiko, Y.V., Phillips, O.R., Weisman, E.F., Wall, D.M., Jo, B., Rasgon, N., 2019. Brain and behavioral correlates of insulin resistance in youth with depression and obesity. Hormones and behavior 108, 73-83.

Smith, E.A., Russell, A., Lorch, E., Banerjee, S.P., Rose, M., Ivey, J., Bhandari, R., Moore, G.J., Rosenberg, D.R., 2003. Increased medial thalamic choline found in pediatric patients with obsessive-compulsive disorder versus major depression or healthy control subjects: a magnetic resonance spectroscopy study. Biological psychiatry 54, 1399-1405.

Sosic-Vasic, Z., Abler, B., Gron, G., Plener, P., Straub, J., 2017. Effects of a brief cognitive behavioural therapy group intervention on baseline brain perfusion in adolescents with major depressive disorder. Neuroreport 28, 348-353.

Steingard, R.J., Renshaw, P.F., Yurgelun-Todd, D., Appelmans, K.E., Lyoo, I.K., Shorrock, K.L., Bucci, J.P., Cesena, M., Abebe, D., Zurakowski, D., Poussaint, T.Y., Barnes, P., 1996. Structural abnormalities in brain magnetic resonance images of depressed children. Journal of the American Academy of Child and Adolescent Psychiatry 35, 307-311.

Straub, J., Brown, R., Malejko, K., Bonenberger, M., Gron, G., Plener, P.L., Abler, B., 2019. Adolescent depression and brain development: evidence from voxel-based morphometry. Journal of psychiatry & neuroscience : JPN 44, 1-9.

Straub, J., Metzger, C.D., Plener, P.L., Koelch, M.G., Groen, G., Abler, B., 2017. Successful group psychotherapy of depression in adolescents alters fronto-limbic resting-state connectivity. Journal of affective disorders 209, 135-139.

Strigo, I.A., Simmons, A.N., Matthews, S.C., Craig, A.D., Paulus, M.P., 2008. Association of major depressive disorder with altered functional brain response during anticipation and processing of heat pain. Archives of general psychiatry 65, 1275-1284.

Strikwerda-Brown, C., Davey, C.G., Whittle, S., Allen, N.B., Byrne, M.L., Schwartz, O.S., Simmons, J.G., Dwyer, D., Harrison, B.J., 2015. Mapping the relationship between subgenual cingulate cortex functional connectivity and depressive symptoms across adolescence. Social cognitive and affective neuroscience 10, 961-968.

Stringaris, A., Vidal-Ribas Belil, P., Artiges, E., Lemaitre, H., Gollier-Briant, F., Wolke, S., Vulser, H., Miranda, R., Penttila, J., Struve, M., Fadai, T., Kappel, V., Grimmer, Y., Goodman, R., Poustka, L., Conrod, P., Cattrell, A., Banaschewski, T., Bokde, A.L., Bromberg, U., Buchel, C., Flor, H., Frouin, V., Gallinat, J., Garavan, H., Gowland, P., Heinz, A., Ittermann, B., Nees, F., Papadopoulos, D., Paus, T., Smolka, M.N., Walter, H., Whelan, R., Martinot, J.L., Schumann, G., Paillere-Martinot, M.L., 2015. The brain's response to reward anticipation and depression in adolescence: dimensionality, specificity, and longitudinal predictions in a community-based sample. The American journal of psychiatry 172, 1215-1223.

Sun, H., Luo, L., Yuan, X., Zhang, L., He, Y., Yao, S., Wang, J., Xiao, J., 2018. Regional homogeneity and functional connectivity patterns in major depressive disorder, cognitive vulnerability to depression and healthy subjects. Journal of affective disorders 235, 229-235.

Tannous, J., Amaral-Silva, H., Cao, B., Wu, M.J., Zunta-Soares, G.B., Kazimi, I., Zeni, C., Mwangi, B., Soares, J.C., 2018. Hippocampal subfield volumes in children and adolescents with mood disorders. Journal of psychiatric research 101, 57-62.

Tao, R., Calley, C.S., Hart, J., Mayes, T.L., Nakonezny, P.A., Lu, H., Kennard, B.D., Tamminga, C.A., Emslie, G.J., 2012. Brain activity in adolescent major depressive disorder before and after fluoxetine treatment. Am J Psychiatry 169, 381-388.

Tymofiyeva, O., Henje Blom, E., Ho, T.C., Connolly, C.G., Lindqvist, D., Wolkowitz, O.M., Lin, J., LeWinn, K.Z., Sacchet, M.D., Han, L.K.M., Yuan, J.P., Bhandari, S.P., Xu, D., Yang, T.T., 2018. High levels of mitochondrial DNA are associated with adolescent brain structural hypoconnectivity and increased anxiety but not depression. Journal of affective disorders 232, 283-290.

Tymofiyeva, O., Yuan, J.P., Huang, C.-Y., Connolly, C.G., Henje Blom, E., Xu, D., Yang, T.T., 2019. Application of machine learning to structural connectome to predict symptom reduction in depressed adolescents with cognitive behavioral therapy (CBT). NeuroImage. Clinical 23, 101914-101914.

van Hoof, M.J., van den Bulk, B.G., Rombouts, S., Rinne-Albers, M.A.W., van der Wee, N.J.A., van, I.M.H., Vermeiren, R., 2017. Emotional face processing in adolescents with childhood sexual abuse-related posttraumatic stress disorder, internalizing disorders and healthy controls. Psychiatry research. Neuroimaging 264, 52-59.

Venta, A., Sharp, C., Patriquin, M., Salas, R., Newlin, E., Curtis, K., Baldwin, P., Fowler, C., Frueh, B.C., 2018. Amygdala-frontal connectivity predicts internalizing symptom recovery among inpatient adolescents. Journal of affective disorders 225, 453-459.

Vilgis, V., Chen, J., Silk, T.J., Cunnington, R., Vance, A., 2014. Frontoparietal function in young people with dysthymic disorder (DSM-5: Persistent depressive disorder) during spatial working memory. Journal of affective disorders 160, 34-42.

Vilgis, V., Vance, A., Cunnington, R., Silk, T.J., 2017. White matter microstructure in boys with persistent depressive disorder. Journal of affective disorders 221, 11-16.

Wohlschlager, A., Karne, H., Jordan, D., Lowe, M.J., Jones, S.E., Anand, A., 2018. Spectral dynamics of resting state fMRI within the ventral tegmental area and dorsal raphe nuclei in medication-free major depressive disorder in young Adults. Frontiers in psychiatry 9, 163.

Wolke, S.A., Mehta, M.A., O'Daly, O., Zelaya, F., Zahreddine, N., Keren, H., O'Callaghan, G., Young, A., Leibenluft, E., Pine, D.S., Stringaris, A., 2019. Modulation of anterior cingulate cortex reward and penalty signalling in medication-naive young-adult subjects with depressive symptoms following acute dose lurasidone. Psychological medicine, 1-13.

Wu, M.J., Wu, H.E., Mwangi, B., Sanches, M., Selvaraj, S., Zunta-Soares, G.B., Soares, J.C., 2015. Prediction of pediatric unipolar depression using multiple neuromorphometric measurements: a pattern classification approach. Journal of psychiatric research 62, 84-91.

Xiao, J., He, Y., McWhinnie, C.M., Yao, S., 2015. Altered white matter integrity in individuals with cognitive vulnerability to depression: a tract-based spatial statistics study. Scientific reports 5, 9738.

Yang, X.R., Langevin, L.M., Jaworska, N., Kirton, A., Lebel, R.M., Harris, A.D., Jasaui, Y., Wilkes, T.C., Sembo, M., Swansburg, R., MacMaster, F.P., 2016. Proton spectroscopy study of the dorsolateral prefrontal cortex in youth with familial depression. Psychiatry and clinical neurosciences 70, 269-277.

Zhang, J., Xiao, J., Zhu, X., Wang, X., Yao, S., 2011a. [Voxel-based morphometry on grey matter concentration of the brain in first-episode, antipsychotic-naive major depressive disorder]. Zhong nan da xue xue bao. Yi xue ban = Journal of Central South University. Medical sciences 36, 307-311.

Zhao, L., Xiao, H., Gao, X., Wang, R., Liu, R., Zhang, P., 2015. 1H-MRS Analysis for prefrontal cortex, hippocampus and thalamus of adolescent patients with depression. Journal of Medical Imaging and Health Informatics 5, 1229-1232.

Zhang, X., Di, X., Lei, H., Yang, J., Xiao, J., Wang, X., Yao, S., Rao, H., 2016. Imbalanced spontaneous brain activity in orbitofrontal-insular circuits in individuals with cognitive vulnerability to depression. Journal of affective disorders 198, 56-63.

Zhang, X., Li, X., Steffens, D.C., Guo, H., Wang, L., 2019. Dynamic changes in thalamic connectivity following stress and its association with future depression severity. Brain Behav 9, e01445-e01445.

Zhang, X., Yao, S., Zhu, X., Wang, X., Zhu, X., Zhong, M., 2012. Gray matter volume abnormalities in individuals with cognitive vulnerability to depression: a voxel-based morphometry study. Journal of affective disorders 136, 443-452.

Zhang, X., Yaseen, Z.S., Galynker, II, Hirsch, J., Winston, A., 2011b. Can depression be diagnosed by response to mother's face? A personalized attachment-based paradigm for diagnostic fMRI. PloS one 6, e27253.

Zhang, X., Zhu, X., Wang, X., Zhu, X., Zhong, M., Yi, J., Rao, H., Yao, S., 2014. First-episode medication-naive major depressive disorder is associated with altered resting brain function in the affective network. PloS one 9, e85241.

Zhong, M., Wang, X., Xiao, J., Yi, J., Zhu, X., Liao, J., Wang, W., Yao, S., 2011. Amygdala hyperactivation and prefrontal hypoactivation in subjects with cognitive vulnerability to depression. Biological psychology 88, 233-242.

Zhu, X., Wang, X., Xiao, J., Liao, J., Zhong, M., Wang, W., Yao, S., 2012. Evidence of a dissociation pattern in resting-state default mode network connectivity in first-episode, treatment-naive major depression patients. Biological psychiatry 71, 611-617.

Zhu, X., Wang, X., Xiao, J., Zhong, M., Liao, J., Yao, S., 2011. Altered white matter integrity in first-episode, treatment-naive young adults with major depressive disorder: a tract-based spatial statistics study. Brain research 1369, 223-229.

Zhu, X., Zhu, Q., Shen, H., Liao, W., Yuan, F., 2017. Rumination and Default Mode Network Subsystems Connectivity in First-episode, Drug-Naive Young Patients with Major Depressive Disorder. Scientific reports 7, 43105.
